# Supplementary material for: NPY-mediated synaptic plasticity in the extended amygdala prioritizes feeding during starvation
Source: Nat Commun. 2024 Jun 27;15:5439. doi: 10.1038/s41467-024-49766-0 (PMC11211344; doi:10.1038/s41467-024-49766-0)
Supplement: Supplementary file 1 — Supplementary Information [file 41467_2024_49766_MOESM1_ESM.pdf]

Supplementary Information

NPY-mediated synaptic plasticity in the extended amygdala prioritizes feeding during starvation

Dodt, S. et al.

Supplementary Fig. 1

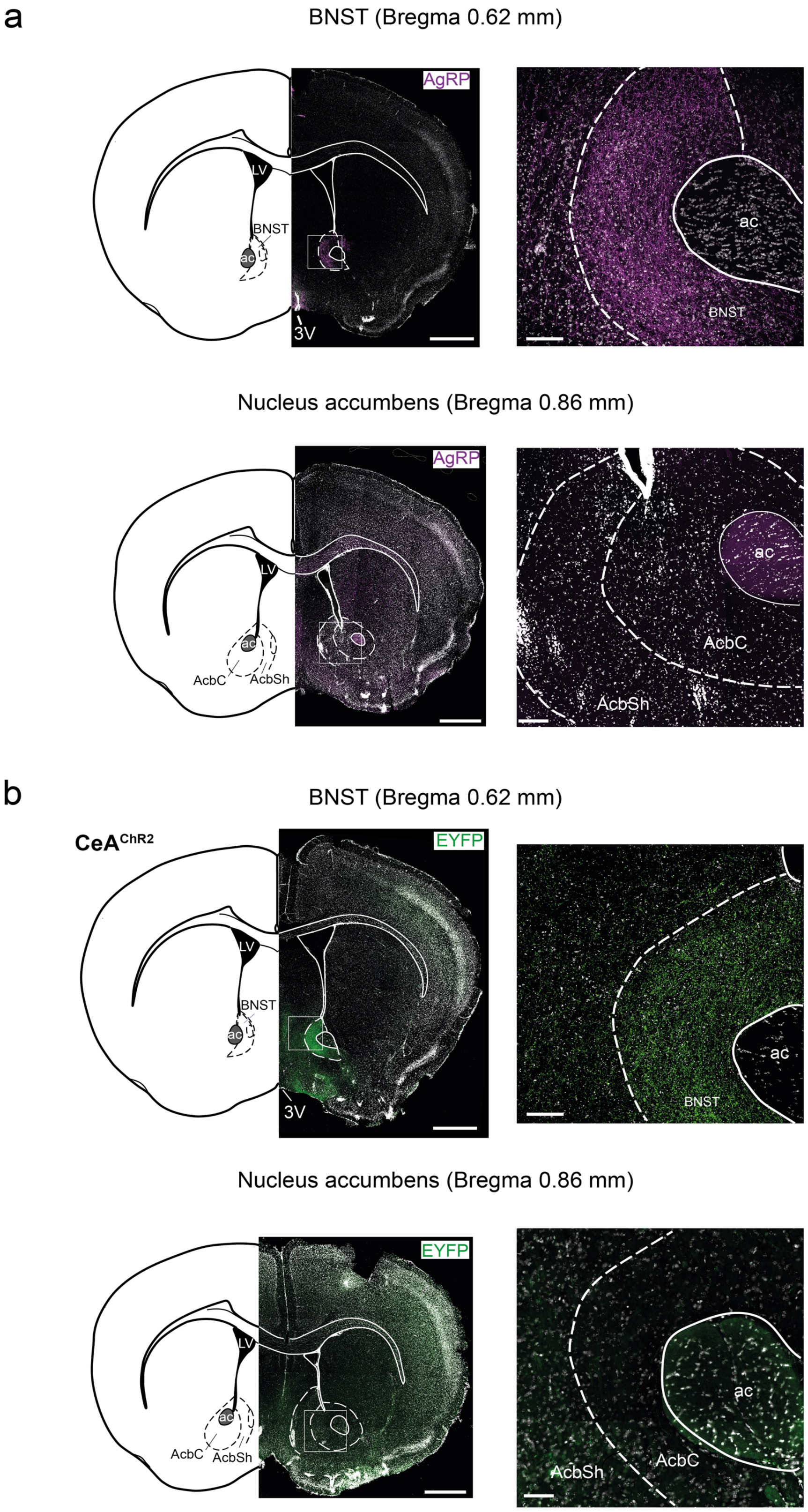

Supplementary Fig. 1: Axonal projections of AgRP neurons and GABAergic CeA neurons in the BNST

(a, b) AgRP and GABAergic CeA neurons innervate the anterior BNST, but not the nucleus accumbens. Representative images showing AgRP and ChR2-EYFP (CeA^{ChR2} mouse) expression.

Scale bars: 1 mm (left), 100 µm (right); AcbC, nucleus accumbens core; AcbSh, nucleus accumbens shell; ac, anterior commissure; 3V, third ventricle; LV, lateral ventricle.

Supplementary Fig. 2

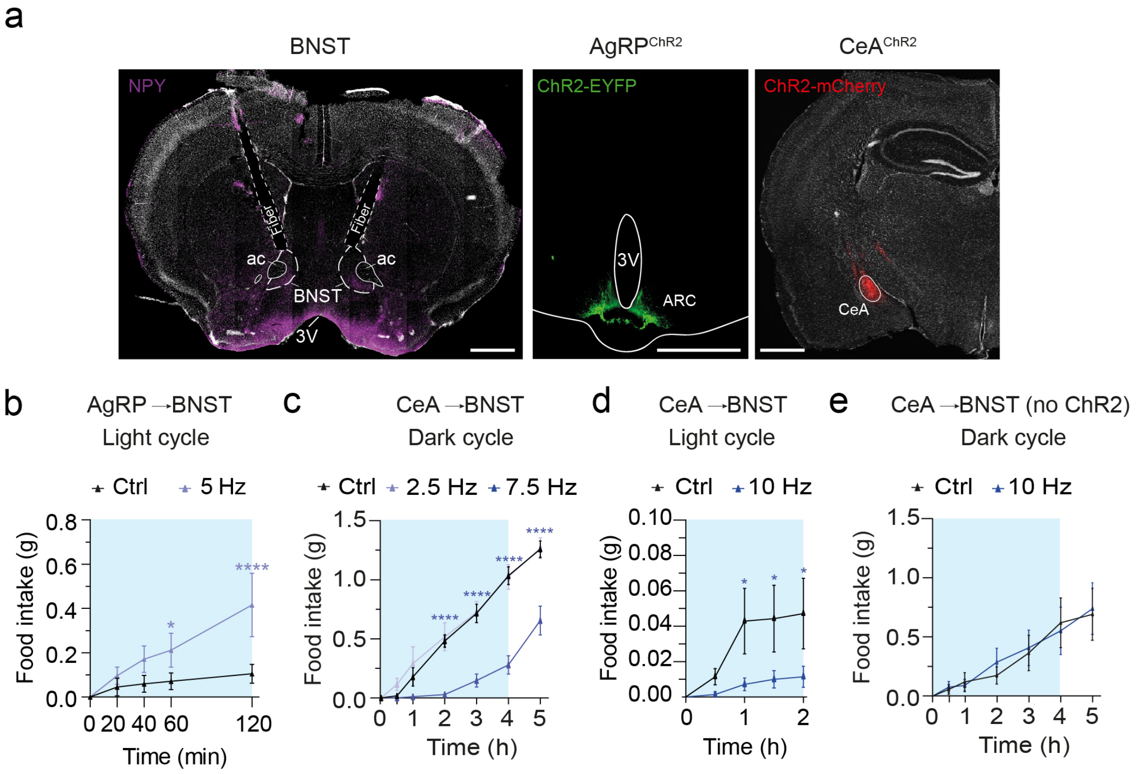

Supplementary Fig. 2: Validation of the optogenetic approach and dose-dependent inhibition of feeding via GABAergic CeA projections in the BNST.

(a) Representative images showing fiber placement above the BNST (left), and ChR2-EYFP expression (green) in the ARC of *Agrp-Ires-Cre; R26-lsl-Chr2-Eyfp* mice and ChR2-mCherry expression (red) in the CeA of *Vgat-Ires-Cre* mice.

(b) Cumulative light-cycle food intake during photostimulation (5 Hz) of AgRP^{ChR2} projections in the BNST compared to no photostimulation (Ctrl; N = 8 animals). Blue box indicates time of photostimulation. *p = 0.0335, ****p < 0.0001 (two-way ANOVA with Šidák post hoc test).

(c) Cumulative dark-cycle food intake during photostimulation (2.5 Hz or 7.5 Hz) of CeA^{ChR2} projections in the BNST compared to no photostimulation (Ctrl; N = 7 animals). ****p < 0.0001 (two-way ANOVA with Šidák post hoc test).

(d) Cumulative light-cycle food intake during photostimulation (10 Hz) of CeA^{ChR2} projections in the BNST compared to no photostimulation (Ctrl; N = 7 animals). *p = 0.0102, *p = 0.0143, *p = 0.0102 (two-way ANOVA with Šidák post hoc test).

(e) Cumulative dark-cycle food intake during photostimulation (10 Hz) of control mice lacking ChR2 expression (N = 5 animals).

Scale bars: 1 mm; ac, anterior commissure; 3V, third ventricle.

All data are presented as mean ± SEM. Asterisks indicate significant differences to the control condition. Source data are provided as a Source Data file.

Supplementary Fig. 3

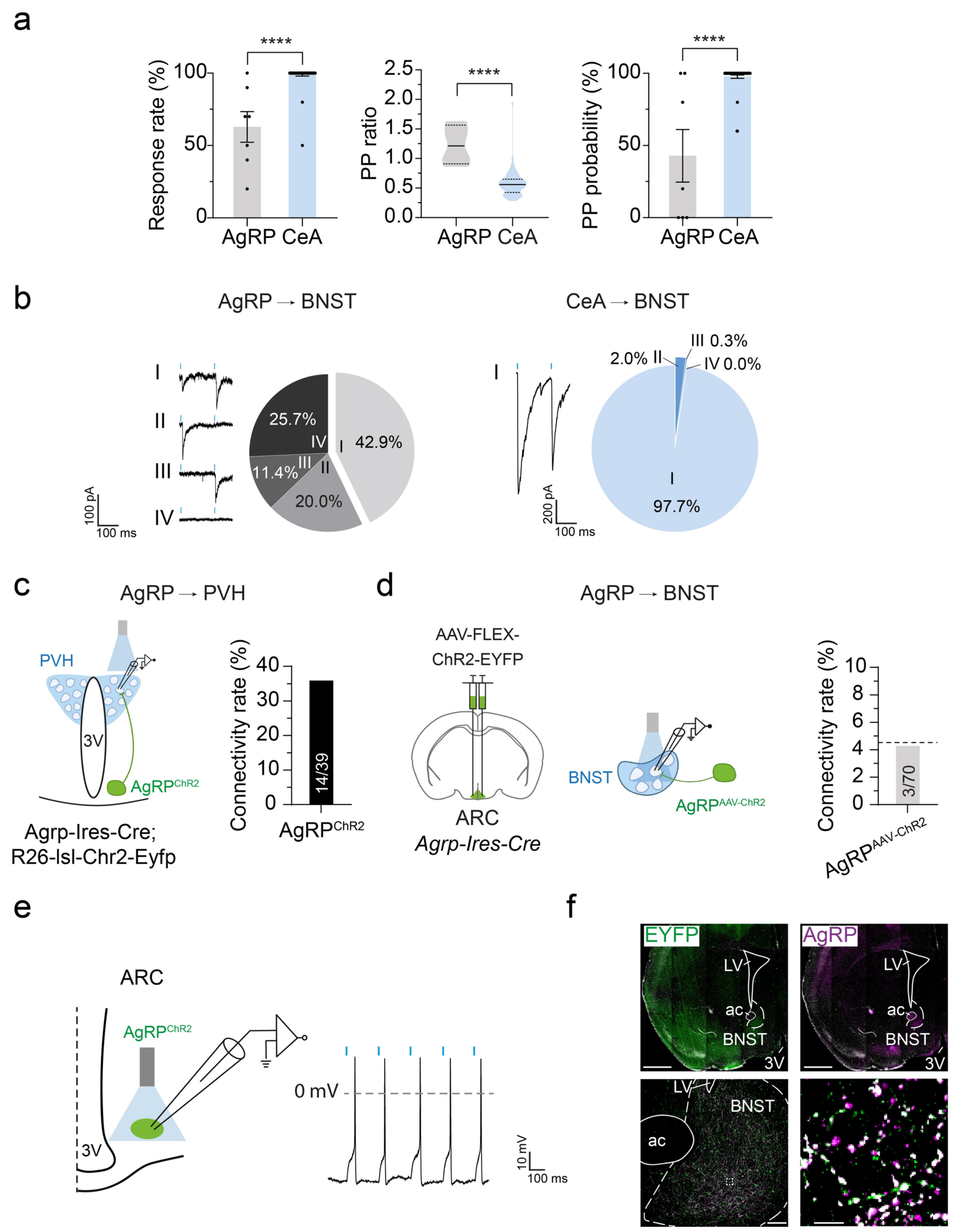

Supplementary Fig. 3: AgRP→BNST and CeA→BNST synapses have distinct properties.

(a) Summaries of ratios of eIPSCs evoked by 10 light pulses (response rate), amplitude of the second eIPSC in relation to the amplitude of the first eIPSC in response to a paired light pulse (250 ms apart; paired pulse (PP) ratio) and probability that both light pulses of a paired pulse will

evoke an eIPSC (PP probability) for AgRP→BNST/CeA→BNST synapses (17/5 brain slices from 13/5 AgRP^{ChR2}/CeA^{ChR2} animals). ****p < 0.0001 (two-sided Mann-Whitney test).

(b) Representative traces from voltage-clamp recordings of BNST neurons illustrating various combinations of eIPSCs in response to paired light pulses in AgRP^{ChR2}/CeA^{ChR2} mice: both light pulses (I), only the first light pulse (II), only the second light pulse (III), or none of the two light pulses (IV) evoked an eIPSC. Summary showing relative distributions of the distinct combinations of eIPSCs evoked by paired light pulses (17/5 brain slices from 13/5 AgRP^{ChR2}/CeA^{ChR2} animals).

(c) Schematic illustration of the approach used for electrophysiological characterization of the AgRP→PVH circuit in AgRP^{ChR2} mice. Summary of the connectivity rate for the AgRP→PVH circuit (3 brain slices from 3 animals).

(d) Schematic showing the virally-mediated expression of ChR2 in AgRP neurons. Summary of the connectivity rate for the AgRP→BNST circuit in AgRP$^{AAV\text{-}ChR2}$ mice (6 brain slices from 4 animals). Dashed lines indicate the connectivity rate to BNST neurons in transgenic AgRP^{ChR2} mice (see Fig. 2c).

(e) Schematic illustration of the approach used for electrophysiological recordings of AgRP neurons expressing AAV-FREX-ChR2-EYFP (AgRP^{ChR2}). Representative trace from a current-clamp recording of an AgRP^{ChR2} neuron exhibiting light-evoked action potentials.

(f) Representative images showing ChR2-EYFP (green) and NPY (magenta) expression in the BNST of an *AgRP-p2a-Dre* mouse that was injected with AAV-FREX-ChR2-EYFP into the ARC.

Scale bars: 1 mm, 100 µm, 5 µm; 3V, third ventricle; ac, anterior commissure; LV, lateral ventricle. Numbers in bars indicate neurons with eIPSCs in relation to all recorded neurons.

Bar graphs represent mean ± SEM. Violin plots represent median ± quartiles. Pie charts represent relative distributions. Source data are provided as a Source Data file.

Schematics in c, d, e were created with Biorender.com released under a Creative Commons Attribution-NonCommercial-NoDerivs 4.0 International license.

Supplementary Fig. 4

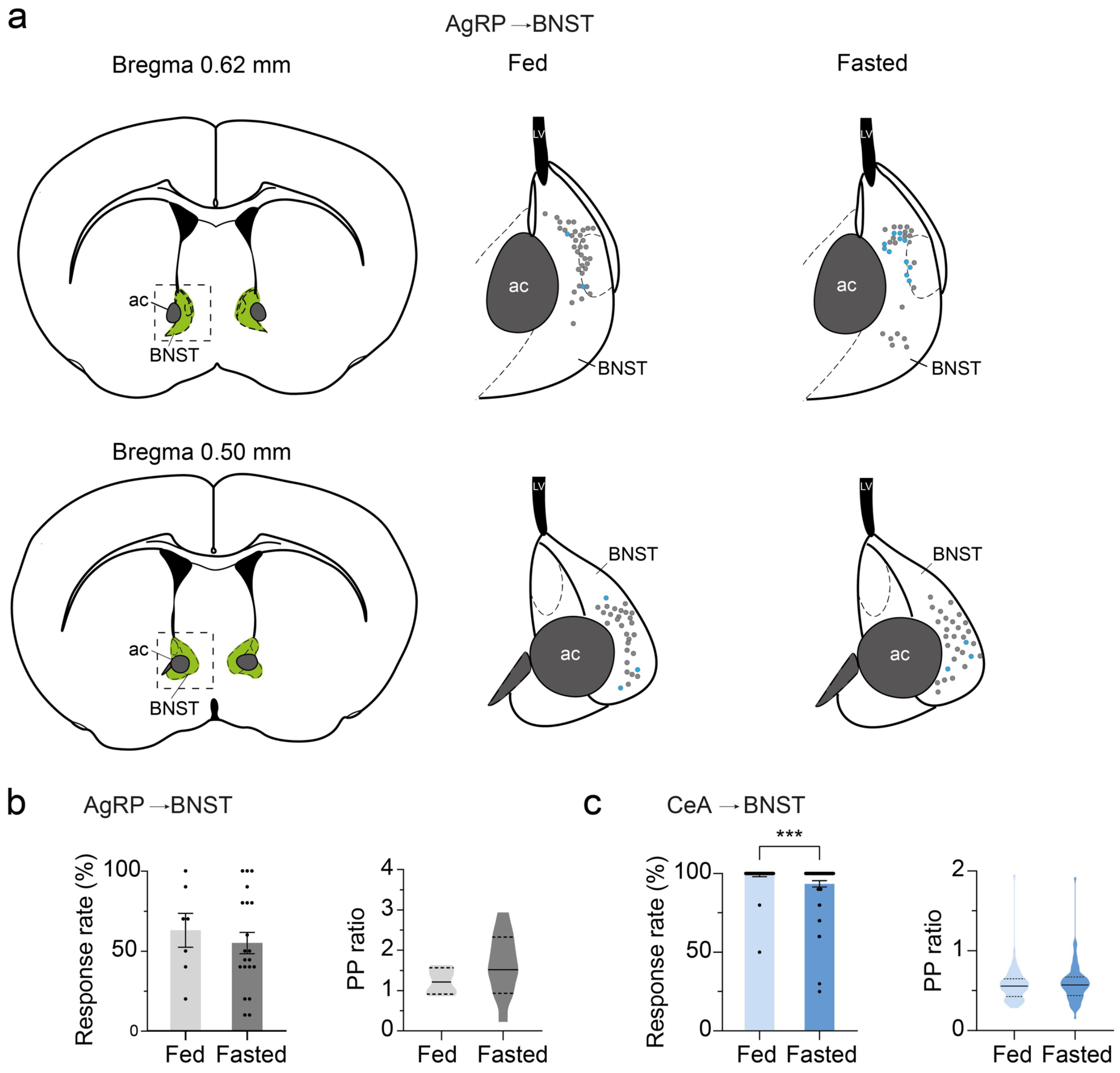

Supplementary Fig. 4: Effects of fasting on AgRP→BNST and CeA→BNST synapses.

(a) Figures illustrating the number and location of BNST neurons receiving GABAergic input from $AgRP^{ChR2}$ neurons (blue circles) under fed (5/65 connected/not connected cells from 6 animals) and fasted (14/61 connected/not connected cells from 5 animals) conditions. Gray circles represent neurons with no eIPSCs.

(b) Summaries of response rates to 10 light pulses and PP ratios of AgRP→BNST synapses in $AgRP^{ChR2}$ mice (16/14 brain slices from 13/11 fed/fasted animals).

(c) Summaries of response rates to 10 light pulses and PP ratios of CeA→BNST synapses in CeA^{ChR2} mice (6/6 brain slices from 5/5 fed/fasted animals). ***$p = 0.0006$ (one-sided Mann-Whitney test).

Bar graphs represent mean ± SEM (b, c). Violin plots represent median ± quartiles (b, c). Source data are provided as a Source Data file.

Supplementary Fig. 5

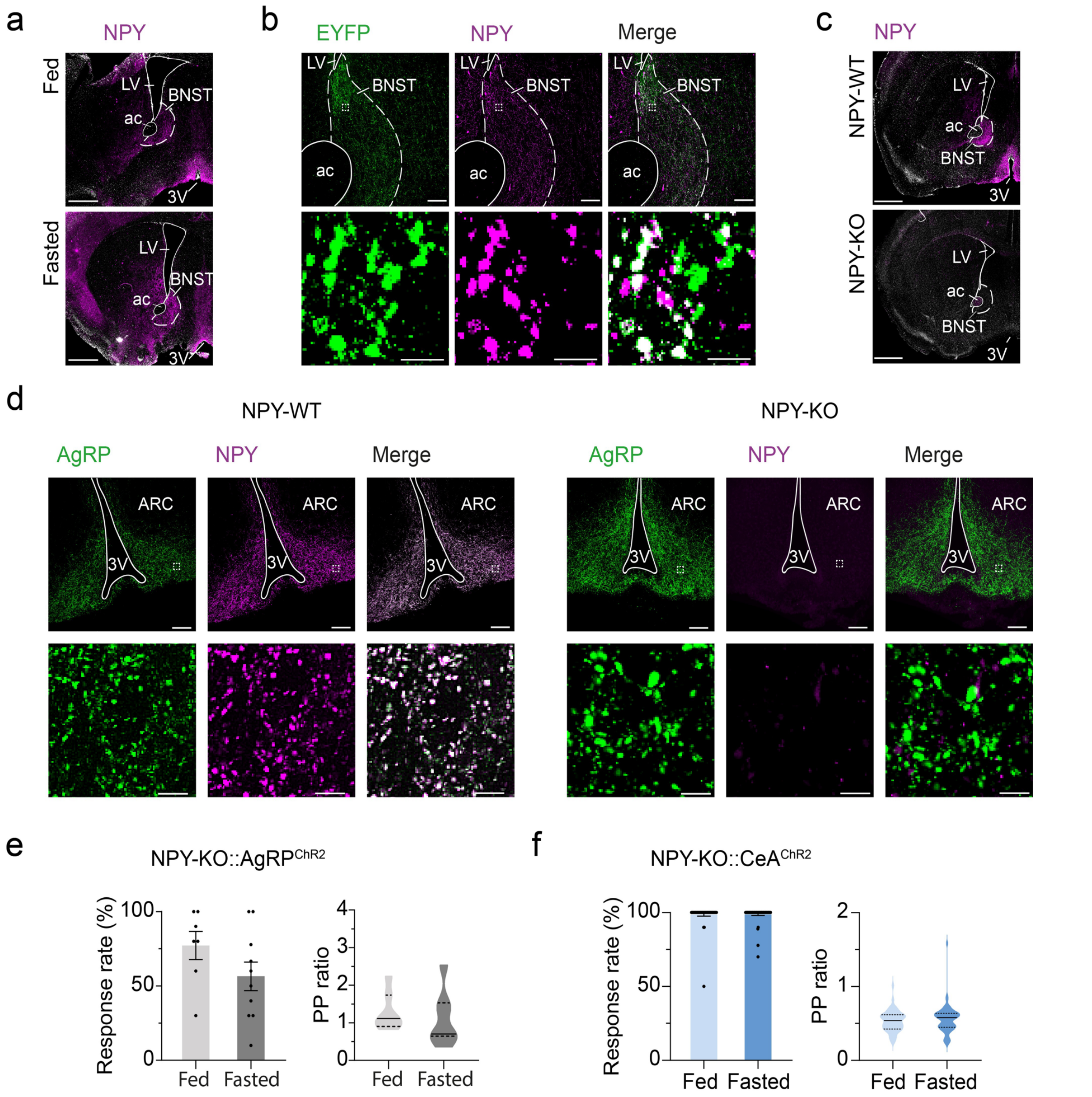

Supplementary Fig. 5: Additional electrophysiological parameters in fed and fasted NPY-KO mice.

(a) Representative images showing expression of NPY (magenta) in the BNST of fed and fasted mice.

(b) Representative images showing ChR2-EYFP (green) and NPY (magenta) expression in (a) the BNST of a fasted AgRP^{ChR2} mouse

(c) Representative images showing NPY (magenta) expression in the BNST of wildtype (NPY-WT) and NPY-deficient (NPY-KO) mice.

(d) Representative images showing AgRP (green) and NPY (magenta) expression in the ARC of NPY-WT and NPY-KO mice.

(e) Summaries of response rates to 10 light pulses and PP ratios of AgRP→BNST synapses in NPY-KO::AgRP^{ChR2} mice (16/17 brain slices from 12/13 fed/fasted animals).

(f) Summaries of response rates to 10 light pulses and PP ratios of CeA→BNST synapses in NPY-KO::CeA^{ChR2} mice (3/4 brain slices from 3/4 fed/fasted animals).

Scale bars: 1 mm (a. c), 100 µm (b, d), 5 µm (b, d); ac, anterior commissure; 3V, third ventricle; LV, lateral ventricle.

Bar graphs represent mean ± SEM (c, d). Violin plots represent median ± quartiles (c, d). Source data are provided as a Source Data file.

Supplementary Fig. 6

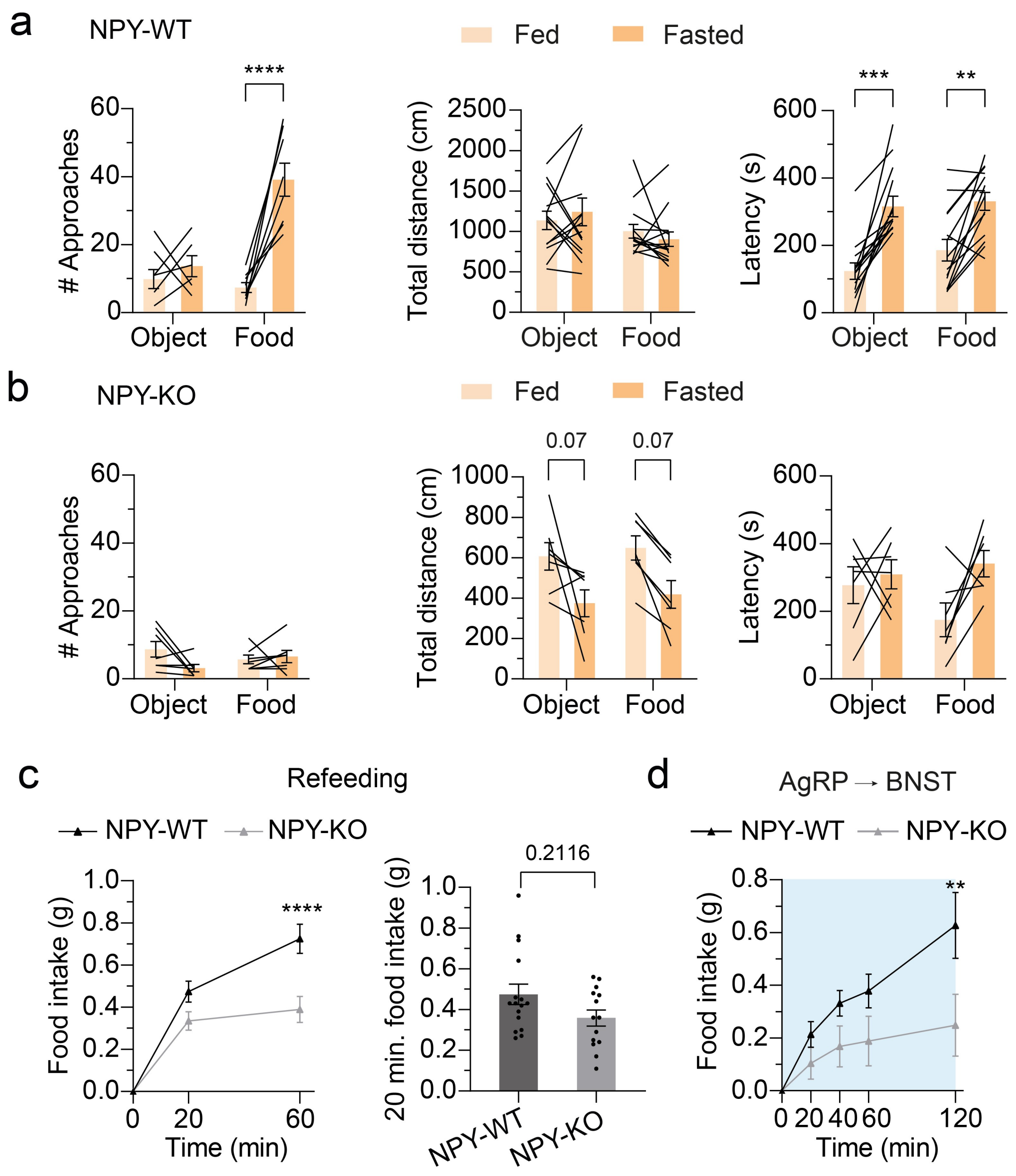

Supplementary Fig. 6: Feeding behavior of NPY-WT and NPY-KO mice.

(a, b) Summaries of the total number of approaches towards the neutral objects and the food pellets located on the open arms of the EOM, the total distance traveled in the EOM, and the latency to enter the open arms of the EOM in fed/fasted wildtype (NPY-WT; a, N = 14 animals) and NPY-deficient (NPY-KO; b, N = 7 animals) mice. **p = 0.0021, ***p = 0.0001, ****p < 0.0001 (two-way ANOVA with Šidák post hoc test).

(c) Cumulative food intake of NPY-WT/NPY-KO mice during refeeding after an overnight fast (N = 16/14 NPY-WT/NPY-KO animals). Total food intake of NPY-WT/NPY-KO mice during 20 min. refeeding after an overnight fast (N = 16/14 NPY-WT/NPY-KO animals). $p = 0.2116$ (two-sided Mann-Whitney test), $^{****}p < 0.0001$ (two-way ANOVA with Šidák post hoc test).

(d) Cumulative food intake of NPY-WT/NPY-KO mice during photostimulation (20 Hz, 1 sec ON/3 sec. OFF) of $AgRP^{ChR2}$ terminals in the BNST (N = 6/5 NPY-WT/NPY-KO animals). Blue box indicates time of photostimulation. $^{**}p = 0.0044$ (two-way ANOVA with Šidák post hoc test).

All data are presented as mean ± SEM. Source data are provided as a Source Data file.

Supplementary Fig. 7

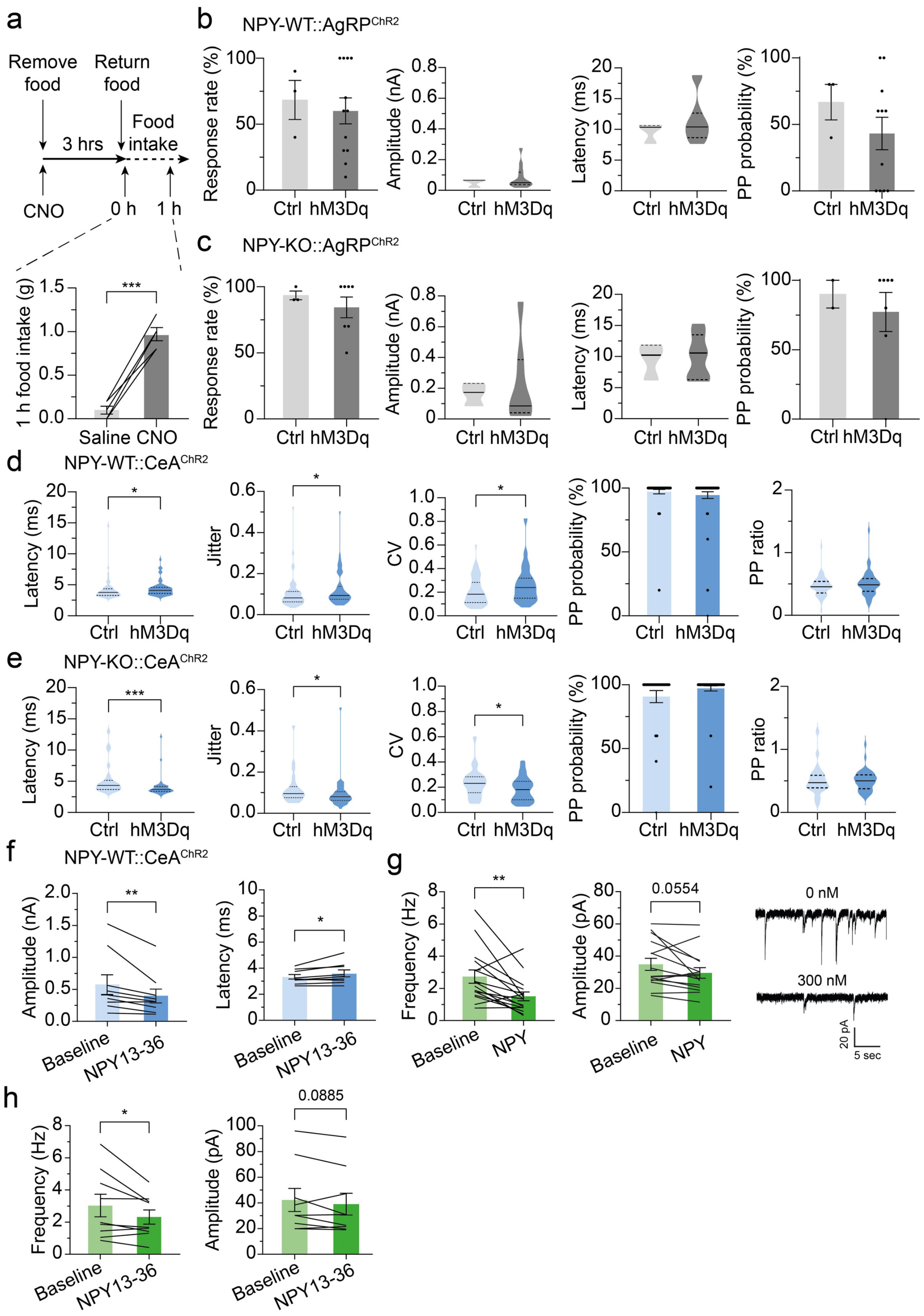

Supplementary Fig. 7: Chemogenetic activation of AgRP neurons is sufficient for inducing the NPY-dependent plasticity of GABAergic synapses in the BNST.

(a) Experimental approach for selective chemogenetic activation of AgRP neurons. Mice were given food access 3 hrs after Saline/CNO administration. Food intake of mice expressing hM3Dq in AgRP neurons (N = 5 animals). ***p = 0.0002 (one-sided paired t-test).

(b, c) Summaries of response rates to 10 light pulses, amplitudes, latencies and PP probabilities of eIPSCs recorded from BNST neurons in NPY-WT:AgRP^{ChR2}/NPY-KO::AgRP^{ChR2} mice with (hM3Dq) or without (Ctrl) expression of hM3Dq in AgRP neurons. Mice were sacrificed 3 hrs following an i.p. injection of CNO (b, NPY-WT::AgRP^{ChR2}: 7/7 brain slices from 5/5 Ctrl/hM3Dq animals; c, NPY-KO::AgRP^{ChR2}: 5/9 brain slices from 4/7 Ctrl/hM3Dq animals).

(d, e) Summaries of latencies, jitter, CVs, PP probabilities and PP ratios of eIPSC recorded from BNST neurons in NPY-WT::CeA^{ChR2}/NPY-KO::CeA^{ChR2} mice with (hM3Dq) or without (Ctrl) expression of hM3Dq in AgRP neurons. Mice were sacrificed 3 hrs following an i.p. injection of CNO (d, NPY-WT::CeA^{ChR2}: 4/5 brain slices from 4/4 Ctrl/hM3Dq animals; e, NPY-KO::CeA^{ChR2}: 3/4 brain slices from 2/4 Ctrl/hM3Dq animals). *p = 0.0139, *p = 0.0481, *p = 0.0152, ***p = 0.0003, *p = 0.0424, (one-sided Mann-Whitney test), *p = 0.0311 (one-sided unpaired t-test).

(f) Summaries of eIPSC amplitudes and latencies recorded from BNST neurons in NPY-WT::CeA^{ChR2} mice before (Baseline) and after (NPY13-36) bath application of the NPY2R agonist NPY13-36 (1 μM; n = 9 cells). **p = 0.0039 (one-sided Wilcoxon test), *p = 0.0221 (one-sided paired t-test).

(g, h) Summaries of frequencies and amplitudes of spontaneous IPSCs (sIPSCs) recorded from BNST neurons in wildtype mice before (Baseline) and after (NPY) bath application of NPY (g; 0.3; μM; n = 16 cells) or the NPY2R agonist NPY13-36 (h; 1 μM; n = 9 cells). Representative traces of a voltage clamp recording illustrating the effect of NPY on the spontaneous inhibitory input of BNST neurons (g). **p = 0.0059 (two-sided paired t-test), p = 0.0554 (two-sided Wilcoxon test), *p = 0.0278, p = 0.0885 (one-sided paired t-test).

Bar graphs represent mean ± SEM (a-g). Violin plots represent median ± quartiles (b, c, d, e). Source data are provided as a Source Data file.

Supplementary Fig. 8

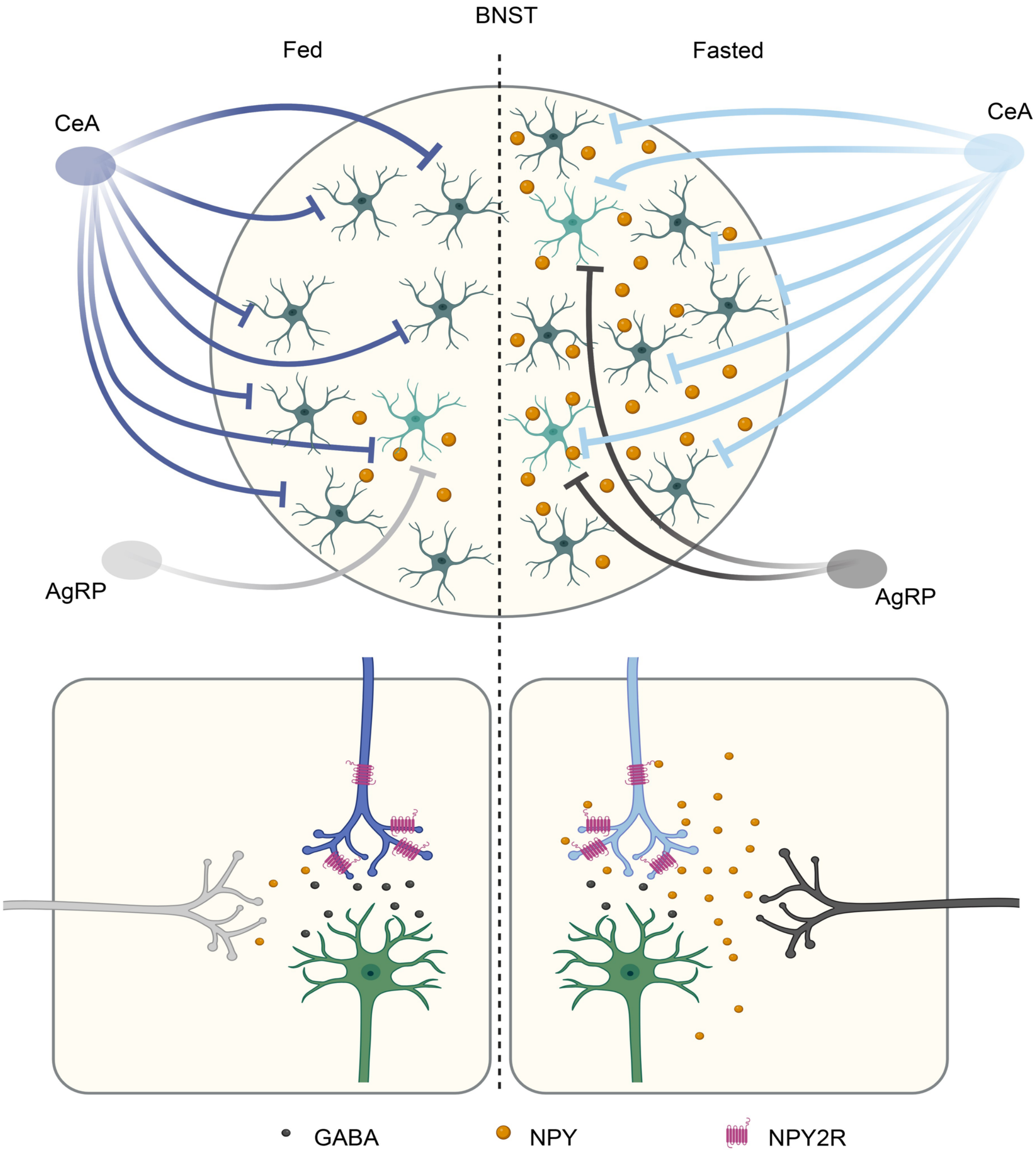

Supplementary Fig. 8: Graphical summary.

We propose the following model: Under fed conditions, connectivity of the GABAergic AgRP→BNST circuit is low while transmission across the GABAergic CeA→BNST synapse is potent and strong. Upon caloric deprivation, connectivity of the AgRP→BNST circuit increases, whereas inhibitory tone emanating from the CeA is markedly reduced. These energy state-dependent, input-specific synaptic changes require NPY from AgRP neurons. The activation of

NPY2Rs on presynaptic terminals mediates the inhibition of GABAergic transmission across the CeA→BNST synapse.

Schematic was created with Biorender.com released under a Creative Commons Attribution-NonCommercial-NoDerivs 4.0 International license.

List of reagents and resources

Reagent or Resource	Source	Identifier
Primary Antibodies		
Goat anti-AgRP	Neuromics	GT15023
Rabbit monoclonal anti-NPY	Cell signaling	D7Y5A
Chicken polyclonal anti-GFP	Abcam	Ab13970
Rat monoclonal anti-mCherry	Invitrogen	M11217
Secondary Antibodies		
Donkey anti-goat Alexa Fluor 488	Invitrogen	A11055
Donkey anti-goat Alexa Fluor 594	Invitrogen	A11058
Donkey anti-chicken Alexa Fluor 488	Invitrogen	A78948
Donkey anti-rabbit Alexa Fluor 594	Invitrogen	A21207
Donkey anti-rat Alexa Fluor 594	Invitrogen	A21209
Chemicals, peptides and recombinant proteins		
Clozapine N-oxide (CNO)	Hello Bio	HB6149
CNQX	Hello Bio	HB0205
D-AP5	Alomone Labs	D-145
Bicuculline	Sigma-Aldrich	14343
Neuropeptide Y (human, rat)	Tocris	1153
Neuropeptide Y (13-36) (human, rat)	Cayman Chemical Company	24714

Ahx[5-24]-NPY	Prof. Dr. A. Beck-Sickinger	-
Experimental models: Organism/strain		
Mouse: *C57B2/6*	Charles River	027
Mouse: *Agrp$^{tm1(cre)Lowl}$ / J*	Jackson Laboratories	012899
Mouse: *AgRP-p2a-Dre*	Prof. Dr. B. B. Lowell	-
Mouse: *Slc32a1$^{tm2(cre)Lowl}$ / J*	Jackson Laboratories	016962
Mouse: *Mc4r$^{tm3.1(cre)Lowl}$ / J*	Jackson Laboratories	030759
Mouse: *Npy1r$^{tm1.1(cre/GFP)Rpa}$ / J*	Jackson Laboratories	030544
Mouse: *B6;126S-Gt(ROSA)26Sor$^{tm32(CAG-COP4*H134R/EYFP)Hze}$*	Jackson Laboratories	012569
Mouse: *B6;129S-Gt(ROSA)26Sor$^{tm66.1(CAG-tdTomato)Hze}$*	Jackson Laboratories	021876
Mouse: *129S-NPY^{tm1Rpa} / J*	Jackson Laboratories	004545
Plasmids/Cloning kits		
5 MLU primer	Eurogentec	-
3 MLU primer	Eurogentec	-

pGEM®-T Easy	Promega	A3600
Ai27 plasmid	Addgene	34630
Ai32 plasmid	Addgene	34880
5 ascChryfp primer	Eurogentec	-
3 ascChryfp primer	Eurogentec	-
Viruses		
AAV9-hSyn-DIO-mCherry	Addgene	50459-AAV9
AAV1-hSyn-ChR2-EYFP	Addgene	26973-AAV1
AAV8-hSyn-ChR2-mCherry	Addgene	26976-AAV8
AAV1-EF1α-FLEX-ChR2-EYFP	Addgene	20298-AAV1
AAV9-hSyn-DIO-hM3Dq-mCherry	Addgene	44361-AAV9
AAV8-CAG-FREX-Chr2-EYFP	Self-made	-
Software and algorithms		
Biorender	Biorender	https://biorender.com/
Illustrator CC	Adobe Systems Inc.	https://www.adobe.com/ products/illustrator/
Image J	Schneider et al., 2012	https://imagej.nih.gov/ij/
pCLAMP 10.7	Molecular Devices	https://www.moleculardevices.com/
Clampfit	Molecular Devices	https://www.moleculardevices.com/
Prism	GraphPad Inc.	https://www.graphpad.com/scientificsoftware/prism/

R	R Foundation	https://www.r-project.org/
VideoMot 3D Analysis V7.01	TSE systems	https://www.tse-systems.com/
LAS X	Leica<br><br>Micorsystems	https://www.leica-microsystems.com/de/produkte/mikroskop-software/p/leica-las-x-ls/downloads/
ZEN 2	Zeiss	https://www.zeiss.com/microscopy/de/produkte/software/zeiss-zen-lite.html
