# Supplementary material for: NPY-mediated synaptic plasticity in the extended amygdala prioritizes feeding during starvation
Source: Nat Commun. 2024 Jun 27;15:5439. doi: 10.1038/s41467-024-49766-0 (PMC11211344; doi:10.1038/s41467-024-49766-0)
Supplement: Supplementary file 3 — Reporting Summary [file 41467_2024_49766_MOESM3_ESM.pdf]

nature portfolio

Corresponding author(s): Henning Fenselau, PhD

Last updated by author(s): May 21, 2024

Reporting Summary

Nature Portfolio wishes to improve the reproducibility of the work that we publish. This form provides structure for consistency and transparency in reporting. For further information on Nature Portfolio policies, see our Editorial Policies and the Editorial Policy Checklist.

Statistics

For all statistical analyses, confirm that the following items are present in the figure legend, table legend, main text, or Methods section.

n/a	Confirmed	
☐	☒	The exact sample size (*n*) for each experimental group/condition, given as a discrete number and unit of measurement
☐	☒	A statement on whether measurements were taken from distinct samples or whether the same sample was measured repeatedly
☐	☒	The statistical test(s) used AND whether they are one- or two-sided<br>*Only common tests should be described solely by name; describe more complex techniques in the Methods section.*
☒	☐	A description of all covariates tested
☐	☒	A description of any assumptions or corrections, such as tests of normality and adjustment for multiple comparisons
☐	☒	A full description of the statistical parameters including central tendency (e.g. means) or other basic estimates (e.g. regression coefficient) AND variation (e.g. standard deviation) or associated estimates of uncertainty (e.g. confidence intervals)
☐	☒	For null hypothesis testing, the test statistic (e.g. *F*, *t*, *r*) with confidence intervals, effect sizes, degrees of freedom and *P* value noted<br>*Give P values as exact values whenever suitable.*
☒	☐	For Bayesian analysis, information on the choice of priors and Markov chain Monte Carlo settings
☒	☐	For hierarchical and complex designs, identification of the appropriate level for tests and full reporting of outcomes
☒	☐	Estimates of effect sizes (e.g. Cohen's *d*, Pearson's *r*), indicating how they were calculated

Our web collection on statistics for biologists contains articles on many of the points above.

Software and code

Policy information about availability of computer code

Data collection	pClamp 10.7 (Molecular Devices, Sunnyvale, USA); VideoMot 3D Analysis V7.01 (TSE systems, Berlin, Germany);LAS X (Leica Microscope), ZEN 2 (Zeiss Microscope)
Data analysis	Clampfit 10.7 (Molecular Devices, Sunnyvale, USA); VideoMot 3D Analysis V7.01 (TSE systems, Berlin, Germany); R (version 4.2.2) - ggplot2 (version 3.4.2) package; ImageJ software (Schneider, Rasband, & Eliceiri, 2012); Prism 9.3.1 (GraphPad) software

For manuscripts utilizing custom algorithms or software that are central to the research but not yet described in published literature, software must be made available to editors and reviewers. We strongly encourage code deposition in a community repository (e.g. GitHub). See the Nature Portfolio guidelines for submitting code & software for further information.

Data

Policy information about availability of data

All manuscripts must include a data availability statement. This statement should provide the following information, where applicable:

- Accession codes, unique identifiers, or web links for publicly available datasets
- A description of any restrictions on data availability
- For clinical datasets or third party data, please ensure that the statement adheres to our policy

No original code was generated in this study. Any additional information required to reanalyze the data reported in this paper is available from the lead contact upon request. No unique reagents were generated in this study. Further information and requests for resources and reagents should be directed and will be fulfilled by the corresponding author.

Research involving human participants, their data, or biological material

Policy information about studies with human participants or human data. See also policy information about sex, gender (identity/presentation), and sexual orientation and race, ethnicity and racism.

Reporting on sex and gender	*Use the terms sex (biological attribute) and gender (shaped by social and cultural circumstances) carefully in order to avoid confusing both terms. Indicate if findings apply to only one sex or gender; describe whether sex and gender were considered in study design; whether sex and/or gender was determined based on self-reporting or assigned and methods used.*<br>*Provide in the source data disaggregated sex and gender data, where this information has been collected, and if consent has been obtained for sharing of individual-level data; provide overall numbers in this Reporting Summary. Please state if this information has not been collected.*<br>*Report sex- and gender-based analyses where performed, justify reasons for lack of sex- and gender-based analysis.*
Reporting on race, ethnicity, or other socially relevant groupings	*Please specify the socially constructed or socially relevant categorization variable(s) used in your manuscript and explain why they were used. Please note that such variables should not be used as proxies for other socially constructed/relevant variables (for example, race or ethnicity should not be used as a proxy for socioeconomic status).*<br>*Provide clear definitions of the relevant terms used, how they were provided (by the participants/respondents, the researchers, or third parties), and the method(s) used to classify people into the different categories (e.g. self-report, census or administrative data, social media data, etc.)*<br>*Please provide details about how you controlled for confounding variables in your analyses.*
Population characteristics	*Describe the covariate-relevant population characteristics of the human research participants (e.g. age, genotypic information, past and current diagnosis and treatment categories). If you filled out the behavioural & social sciences study design questions and have nothing to add here, write "See above."*
Recruitment	*Describe how participants were recruited. Outline any potential self-selection bias or other biases that may be present and how these are likely to impact results.*
Ethics oversight	*Identify the organization(s) that approved the study protocol.*

Note that full information on the approval of the study protocol must also be provided in the manuscript.

Field-specific reporting

Please select the one below that is the best fit for your research. If you are not sure, read the appropriate sections before making your selection.

☒ Life sciences ☐ Behavioural & social sciences ☐ Ecological, evolutionary & environmental sciences

For a reference copy of the document with all sections, see nature.com/documents/nr-reporting-summary-flat.pdf

Life sciences study design

All studies must disclose on these points even when the disclosure is negative.

Sample size	No statistical method was used to predetermine sample size.
Data exclusions	For post hoc analysis of virus expression/optic fiber placement, brain slices of electrophysiological/in vivo experiments were collected and fixed in PFA-PBS before they were washed and stored at 4°C in PBS.
Replication	For all experiments, the number of replicates is mentioned in detail in the legends of figures. All data are reproducible.
Randomization	In general, experimental mice were randomly assigned to their experimental groups. For in vivo experiments, mice were evenly split into different groups and experiments were repeated until each mouse had performed every possible combination of physiological condition/ stimulus.
Blinding	Blinding methods were not used.

Reporting for specific materials, systems and methods

We require information from authors about some types of materials, experimental systems and methods used in many studies. Here, indicate whether each material, system or method listed is relevant to your study. If you are not sure if a list item applies to your research, read the appropriate section before selecting a response.

Materials & experimental systems

n/a	Involved in the study
☐	☒ Antibodies
☒	☐ Eukaryotic cell lines
☒	☐ Palaeontology and archaeology
☐	☒ Animals and other organisms
☒	☐ Clinical data
☒	☐ Dual use research of concern
☒	☐ Plants

Methods

n/a	Involved in the study
☒	☐ ChIP-seq
☒	☐ Flow cytometry
☒	☐ MRI-based neuroimaging

Antibodies

Antibodies used

Goat anti-AgRP (Neuromics; GT15023)
Rabbit anti-NPY (Cell signaling; D7Y5A)
Chicken anti-GFP (Abcam; Ab13970)
Rat anti-mCherry (Invitrogen; M11217)
Donkey anti-goat Alexa Fluor 488 (Invitrogen; A11055)
Donkey anti-goat Alexa Fluor 594 (Invitrogen; A11058)
Donkey anti-chicken Alexa Fluor 488 (Invitrogen; A78948)
Donkey anti-rabbit Alexa Fluor 594 (Invitrogen; A21207)
Donkey anti-rat Alexa Fluor 594 (Invitrogen; A21209)

Validation

All antibodies listed above were previously validated using Western Blot. Please refer to the manufacturers' websites for details.

Animals and other research organisms

Policy information about studies involving animals; ARRIVE guidelines recommended for reporting animal research, and Sex and Gender in Research

Laboratory animals

Mice were monitored for health status daily, housed at 22 – 24°C on a 12 h light/12 h dark cycle, and had ad libitum access to water and to a standard rodent chow diet (ssniff®, V1154) unless food was withdrawn for specific experimental purposes.
Agrp-Ires-Cre (JAX# 012899), Vgat-Ires-Cre (JAX# 016962), Mc4r-t2a-Cre (JAX# 030759), Npy1r-Cre (JAX# 030544), R26-lsl-Chr2-Eyfp (JAX# 012569), R26-lsl-tdTomato (JAX# 021876) and Npy-KO (JAX# 004545) were previously described and purchased from Jackson Laboratories. Agrp-p2a-Dre mice were previously described and kindly provided by Dr. Bradford B. Lowell. C57Bl/6 mice were purchased from Charles River (Strain code: 027). All transgenic mice were bred to C57Bl/6 mice for maintenance. Double transgenic animals and control mice were generated by crossing Cre-expressing mice with Rosa26 transgenic or knockout mice. Triple transgenic mice were generated by crossing double transgenic mice with Agrp-p2a-Dre or Npy-KO mice.

Wild animals

The study did not involve wild animals.

Reporting on sex

For all experiments, we aimed to include the same number of animals of both sexes. Aside from natural differences, we did not observe any obvious differences in electrophysiological parameters of the investigated neurons, in the expression profile of the investigated neuropeptides, or in the anxiety-related behavior of the mice.

Field-collected samples

This study did not include samples collected from the field.

Ethics oversight

All experimental procedures were conducted in compliance with protocols approved by local government authorities (Bezirksregierung Köln).

Note that full information on the approval of the study protocol must also be provided in the manuscript.

Plants

Seed stocks	*Report on the source of all seed stocks or other plant material used. If applicable, state the seed stock centre and catalogue number. If plant specimens were collected from the field, describe the collection location, date and sampling procedures.*
Novel plant genotypes	*Describe the methods by which all novel plant genotypes were produced. This includes those generated by transgenic approaches, gene editing, chemical/radiation-based mutagenesis and hybridization. For transgenic lines, describe the transformation method, the number of independent lines analyzed and the generation upon which experiments were performed. For gene-edited lines, describe the editor used, the endogenous sequence targeted for editing, the targeting guide RNA sequence (if applicable) and how the editor was applied.*
Authentication	*Describe any authentication procedures for each seed stock used or novel genotype generated. Describe any experiments used to assess the effect of a mutation and, where applicable, how potential secondary effects (e.g. second site T-DNA insertions, mosiacism, off-target gene editing) were examined.*
